# A case report of pegcetacoplan use for a pregnant woman with paroxysmal nocturnal hemoglobinuria

**DOI:** 10.1016/j.rpth.2024.102435

**Published:** 2024-05-06

**Authors:** Wei Du, Lin Mei

**Affiliations:** 1 Department of Pathology, University of Pennsylvania, Philadelphia, Pennsylvania, USA; 2 Department of Hematology and Oncology, University of Pennsylvania, Philadelphia, Pennsylvania, USA

**Keywords:** case reports, eculizumab, paroxysmal nocturnal hemoglobinuria (PNH), pegcetacoplan, pregnancy

## Abstract

**Background:**

Paroxysmal nocturnal hemoglobinuria (PNH), a rare hematologic disease, is associated with high maternal and fetal mortality rates. Only 1 medication approved for PNH, the complement component 5 inhibitor eculizumab, has published evidence of use during pregnancy.

**Key Clinical Question:**

What were the circumstances and outcomes of the first use of pegcetacoplan, a complement component 3 inhibitor, by a pregnant woman with PNH?

**Clinical Approach:**

The patient, with a history of 2 miscarriages and a suboptimal response to eculizumab, had hematologic improvement after switching to pegcetacoplan. She continued pegcetacoplan throughout her pregnancy. At gestational week 30, she developed abruptio placentae and breakthrough hemolysis. She delivered a normal-appearing male infant via emergency cesarean section. The breakthrough hemolysis resolved quickly with short-term intensive pegcetacoplan dosing and add-on eculizumab. To date, her laboratory values remain normal, and she has had no thromboembolic events; her son has not demonstrated growth defects.

**Conclusion:**

This is the first report of pegcetacoplan treatment for PNH throughout pregnancy. The mother recovered promptly from breakthrough hemolysis that prompted an emergency delivery. Her son, who was born prematurely but healthy, has developed normally.

## Essentials

• Paroxysmal nocturnal hemoglobinuria (PNH) is dangerous during and after pregnancy.
• This is the first reported case of pegcetacoplan treatment for a pregnant woman with PNH.
• A woman with PNH and a history of miscarriage became pregnant during pegcetacoplan treatment.
• She recovered from breakthrough hemolysis after delivering a healthy but premature baby boy.

## 1 Introduction

Paroxysmal nocturnal hemoglobinuria (PNH)—a rare hematologic disease characterized by intravascular hemolysis, thrombophilia, and bone marrow dysfunction—significantly increases pregnancy complications and is associated with high rates of morbidity and mortality for both mother and infant [[1], [2], [3], [4], [5]]. Pregnant women with PNH face maternal mortality rates as high as 21%, mostly from postpartum thrombotic complications, and fetal mortality rates of up to 9% [[3], [4], [5]]. Consequently, women with PNH have often been advised to avoid pregnancy [1,6]. Additional obstacles to successful pregnancy in women with PNH include the risks for worsening of PNH-related anemia, thromboembolic events, and intravascular hemolysis during pregnancy [7], with thromboembolic risks extending into the postpartum period [3]. Fortunately, data are becoming available suggesting that eculizumab, the first complement component 5 (C5) inhibitor approved for patients with PNH [8], can improve pregnancy outcomes for women with PNH and their infants [6,7]. There are no reports of pregnancy outcomes for women with PNH treated with the next-generation C5 inhibitor ravulizumab, which was approved for the treatment of PNH in 2018 [2,9].

Pegcetacoplan, the first PNH therapy targeting proximal complement C3, has demonstrated superior efficacy compared with eculizumab in patients with PNH who had an incomplete response to eculizumab [10]. No safety data for pegcetacoplan in pregnant women with PNH are currently available. Here we report the successful delivery of a premature, healthy infant by a woman who received pegcetacoplan treatment throughout pregnancy.

## 2 Case Report

Our patient was diagnosed with PNH in 2018 at 33 years of age. She began treatment with intravenous eculizumab shortly after diagnosis, receiving 600 mg weekly for 4 doses, followed by a maintenance dosage of 900 mg every 2 weeks. Her outcomes were suboptimal. She required red blood cell transfusions approximately every 2 to 4 weeks due to intermittent breakthrough hemolysis observed as lactate dehydrogenase concentrations of approximately 300 U/L (upper limit of normal: 192 U/L), bilirubin concentrations of approximately 1 to 2 mg/dL, and frequent decreases in hemoglobin concentration from the baseline of 8 g/dL to <7 g/dL (Figure). Unfortunately, she had 2 miscarriages while receiving eculizumab, the first at 12 weeks’ gestation and the second at 24 weeks, despite an eculizumab dosage increase to 1200 mg once every 2 weeks during the second trimester of her second pregnancy to prevent further hemolysis.

**Figure:**
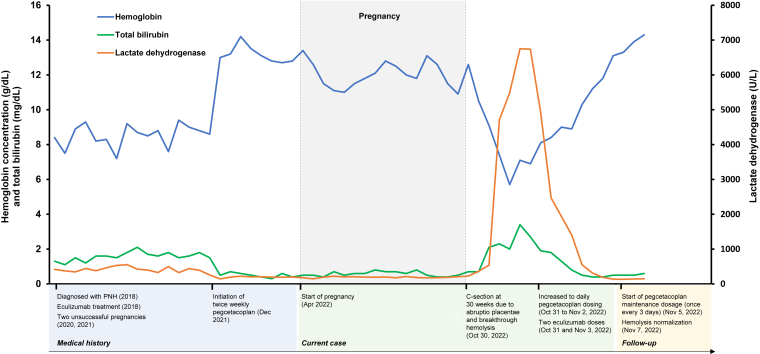
Laboratory results in a woman with paroxysmal nocturnal hemoglobinuria (PNH) before, during, and after pregnancy.

Due to the previous miscarriages and suboptimal PNH control, while receiving eculizumab, the patient switched to subcutaneous (s.c.) pegcetacoplan 1080 mg twice weekly. During the transition to pegcetacoplan, she received pegcetacoplan 1080 mg twice weekly for 4 weeks while continuing eculizumab treatment, followed by pegcetacoplan monotherapy at 1080 mg twice weekly, a protocol used in the phase 3 PEGASUS trial of pegcetacoplan in eculizumab-experienced patients with PNH [10]. She adhered very well to this treatment, and her hemoglobin concentration increased substantially from 8.1 g/dL with eculizumab to 12.6 g/dL approximately 2 weeks after beginning pegcetacoplan (Figure). In April 2022, our patient became pregnant. We discussed with our patient that use of pegcetacoplan during pregnancy had the potential for harm because pegcetacoplan had not been tested during pregnancy and had the potential to cross the placenta. She understood these risks. She continued pegcetacoplan treatment at the dosage she was receiving before pregnancy. Low-molecular-weight heparin (enoxaparin 40 mg s.c. daily) was added to pegcetacoplan in her second trimester; the enoxaparin dosage was later increased to 70 mg daily to account for increased body mass. The patient’s hemoglobin concentration was well maintained with this combination (Figure).

At week 30 of gestation, our patient developed abruptio placentae, and laboratory results showed breakthrough hemolysis (Figure). She required an emergency cesarean section and delivered a normal-appearing male infant. In the intensive care unit, she received intensified treatment for the breakthrough hemolysis. The pegcetacoplan dosage was increased to 1080 mg s.c. once daily for 3 consecutive days. To provide additional complement inhibition, a 600 mg dose of eculizumab was administered at the time of the breakthrough hemolysis, and a second 600 mg dose was given 3 days later. The postpartum breakthrough hemolysis was quickly reversed, and the patient recovered from the breakthrough hemolysis within 1 week. No further eculizumab treatment was given, and she was discharged from the hospital after 1 week, receiving maintenance pegcetacoplan at a dosage of 1080 mg s.c. every 3 days [11]. In a clinic visit 1 week after discharge, her hemoglobin concentration was 13 g/dL, and her lactate dehydrogenase and haptoglobin concentrations had normalized. She also received the standard prophylactic treatment of enoxaparin for 6 weeks after delivery. Her hematologic laboratory values were within normal ranges at her follow-up visits through 6 months postpartum. To date, the mother has not developed any thromboembolic events. Her son has not demonstrated any growth defects, and there have been no other concerns. Pegcetacoplan concentrations in the placenta and breast milk could not be obtained, and the patient did not breastfeed.

## 3 Discussion

To our knowledge, this is the first case of pegcetacoplan therapy throughout pregnancy in a woman with PNH. Our patient gave birth to a healthy, albeit premature, infant. She had a history of 2 failed pregnancies and residual PNH symptoms while receiving eculizumab therapy. Our patient responded well to pegcetacoplan treatment before her pregnancy and during the first and second trimesters. Although she developed complications at gestational week 30, her premature infant was delivered safely, and the breakthrough hemolysis she experienced was quickly reversed by briefly using pegcetacoplan and eculizumab for simultaneous proximal and distal complement pathway inhibition [12]. This resolution of breakthrough hemolysis supports the feasibility of dual complement blockade to treat breakthrough hemolysis in pregnant women with PNH. To date, the mother has not developed any thromboembolic events, and her son has not demonstrated any growth defects.

Historically, pregnancy for women with PNH was associated with high rates of maternal and fetal mortality. The death rate for pregnant women with PNH was between 8% and 21% [[3], [4], [5]], and the fetal death rate ranged from 4% to 9% [[2], [3], [4], [5]]. Emerging evidence indicates more favorable outcomes when pregnant women with PNH receive eculizumab treatment. Real-world data from 75 pregnancies in 61 women with PNH, obtained from surveys given to physicians in the PNH Interest Group and the International PNH Registry, showed no maternal deaths when eculizumab was used during pregnancy [6]. These 75 pregnancies resulted in 69 live births, 6 (8%) miscarriages, and 3 (4%) stillbirths [6]. Likewise, in a retrospective analysis of medical records from the United Kingdom, there were no maternal deaths among 21 women with PNH who received eculizumab during 37 pregnancies [7]. Of these 37 pregnancies, 31 (84%) resulted in a live birth, 3 (8%) ended in miscarriage, and 2 (5%) in fetal death; there was 1 (3%) ectopic pregnancy.

Despite these encouraging findings, both real-world analyses reported high rates of premature birth (26%-29%) [6,7] and a need for increased eculizumab dosages during pregnancy in many patients [6,7]. The current case, in which the baby was born at 30 weeks of gestation and the mother required increased pegcetacoplan dosing, is in line with the findings for eculizumab. The need for an emergency cesarean section after abruptio placentae and maternal breakthrough hemolysis emphasizes that pregnancies in women with PNH must be managed carefully by multiple specialists because these patients have an increased risk of complications, including breakthrough hemolysis during pregnancy and thromboembolic events postpartum [[1], [2], [3]]. At the advice of a PNH expert, our patient received intensive treatment consisting of daily pegcetacoplan s.c. dosing for 3 days and 2 eculizumab doses to control the breakthrough hemolysis. We used this dual blockade approach because the hemolysis occurred during pegcetacoplan treatment, which could indicate that proximal complement inhibition had been bypassed. This was followed by maintenance pegcetacoplan at an increased dose of 1080 mg s.c. every 3 days, as described by Griffin et al. [11] for pegcetacoplan-treated patients with breakthrough hemolysis. Our patient received prophylactic enoxaparin throughout her pregnancy to decrease the risk of thromboembolic events. Although no specific guidelines exist, this precautionary treatment aligns with the anticoagulant use previously reported for eculizumab-treated pregnant women [2,6,7].

The key strengths of this report are its novelty and its encouraging outcome; this is the first case study documenting the use of pegcetacoplan for PNH throughout pregnancy, and both mother and child remain healthy. Our report is limited in that it is only 1 example, so it cannot be readily extended to a wider population. In addition, data about the pegcetacoplan concentrations in the blood and breast milk of the mother and the blood of her infant are not available. Such data should be obtained in future cases to further inform the use of pegcetacoplan in pregnancy. Additional long-term data regarding postpartum thromboembolic events in the mother would also be valuable.

## 4 Conclusion

This is the first report of an infant born to a woman with PNH who received pegcetacoplan throughout her pregnancy. The infant was premature but healthy and without anomalies. The breakthrough hemolysis experienced by the mother during delivery was promptly resolved with short-term intensive pegcetacoplan dosing and add-on eculizumab for dual complement inhibition. This report provides an example of an effective pegcetacoplan treatment regimen during and after a successful pregnancy and can be used to guide subsequent research and real-world treatment decisions.
